# PET imaging of patients with non-small cell lung cancer employing an EGF receptor targeting drug as tracer

**DOI:** 10.1038/bjc.2011.493

**Published:** 2011-11-17

**Authors:** A A Memon, B Weber, M Winterdahl, S Jakobsen, P Meldgaard, H H T Madsen, S Keiding, E Nexo, B S Sorensen

**Affiliations:** 1 Department of Clinical Biochemistry, Aarhus University Hospital, Norrebrogade 44, 8000 Aarhus C, Denmark; 2 Department of Oncology, Aarhus University Hospital, Norrebrogade 44, Aarhus, Denmark; 3 PET Centre, Aarhus University Hospital, Norrebrogade 44, Aarhus, Denmark; 4 Department of Radiology, Aarhus University Hospital, Norrebrogade 44, Aarhus, Denmark

**Keywords:** erlotinib, EGFR, tarceva, lung cancer, PET imaging

## Abstract

**Background:**

We have previously developed ^11^C-erlotinib as a new positron emission tomography (PET) tracer and shown that it accumulates in epidermal growth factor receptor (EGFR)-positive lung cancer xenografts in mice. Here, we present a study in patients with non-small cell lung cancer (NSCLC) investigating the feasibility of ^11^C-erlotinib PET as a potential method for the identification of lung tumours accumulating erlotinib.

**Methods:**

Thirteen patients with NSCLC destined for erlotinib treatment were examined by contrast-enhanced computed tomography (CT), ^11^C-erlotinib PET/low-dose CT and ^18^F-fluoro-2-deoxy-D-glucose (^18^F-FDG) PET/low-dose CT before start of the erlotinib treatment. After 12 weeks treatment, they were examined by ^18^F-FDG PET/contrast-enhanced CT for the assessment of clinical response.

**Results:**

Of the 13 patients included, 4 accumulated ^11^C-erlotinib in one or more of their lung tumours or lymph-node metastases. Moreover, ^11^C-erlotinib PET/CT identified lesions that were not visible on ^18^F-FDG PET/CT. Of the four patients with accumulation of ^11^C-erlotinib, one died before follow-up, whereas the other three showed a positive response to erlotinib treatment. Three of the nine patients with no accumulation died before follow-up, four showed progressive disease while two had stable disease after 12 weeks of treatment.

**Conclusion:**

Our data show a potential for ^11^C-erlotinib PET/CT for visualizing NSCLC lung tumours, including lymph nodes not identified by ^18^F-FDG PET/CT. Large clinical studies are now needed to explore to which extent pre-treatment ^11^C-erlotinib PET/CT can predict erlotinib treatment response.

Lung cancer is one of the leading causes of cancer deaths worldwide (Parkin *et al*, 2005) and the treatment response and clinical outcome of the disease are difficult to predict. Recently, new treatment strategies targeting the epidermal growth factor receptor (EGFR) have been developed. The EGFR is one of the most frequently overexpressed proteins in various cancers including lung cancer, and signalling through this receptor is related to tumour progression and resistance to most treatments (Rusch *et al*, 1993; Fontanini *et al*, 1998; Ciardiello and Tortora, 2008). Therefore, the EGFR has become an attractive target for cancer treatment. The two most commonly used tyrosine kinase inhibitors targeting EGFR are gefitinib (Iressa, ZD1839) and erlotinib (Tarceva, OSI-774). Gefitinib and erlotinib are tailored drugs that compete with adenosine triphosphate (ATP) for the ATP binding site on the EGFR and thereby prevent phosphorylation and activation of downstream signalling molecules involved in cell proliferation and tumour growth. Gefitinib was the first EGFR inhibitor approved for treatment of advanced non-small cell lung cancer (NSCLC); however, clinical trials using gefitinib did not show significant improvement in survival (Comis, 2005). In contrast, trials with erlotinib have demonstrated prolonged progression-free survival and improved survival of patients with advanced NSCLC (Shepherd *et al*, 2004). Erlotinib was also superior to placebo with respect to quality of life (Cohen *et al*, 2005). Nevertheless, overall response rates have been relatively low in studies that have examined all NSCLC patients collectively (Shepherd *et al*, 2005), indicating that not all lung cancer patients are suitable for erlotinib treatment and that the treatment should only be given to selected patients. Various parameters have been used to classify patients who respond to erlotinib, such as type of tumour, smoking history, gender, and ethnicity, but none of these parameters had significant impact on survival (Fukuoka *et al*, 2003; Perez-Soler *et al*, 2004). Patients with tumours expressing high amounts of the EGFR had an improved response to treatment with erlotinib (Shepherd *et al*, 2005) and the presence of specific mutations around the ATP binding domain of the receptor was found to increase the response to gefitinib treatment (Lynch *et al*, 2004; Paez *et al*, 2004). Determination of the EGFR expression and the presence of mutations require a tumour biopsy, which is not possible to collect in all situations. Thus, non-invasive methods are needed that can identify the subset of patients who are most likely to benefit from erlotinib treatment.

Positron emission tomography (PET) is a 3-dimensional imaging technique that uses isotope-labelled tracers that decay with the emission of a positron, it is used for non-invasive assessment of biochemical and physiological processes *in vivo*. In the present study, we used 2-[^18^F]fluoro-2-deoxy-D-glucose (^18^F-FDG) for visualization of the higher glucose metabolism of tumour tissue compared with surrounding tissue (Gambhir, 2002; Jerusalem *et al*, 2003; Rohren *et al*, 2004) and ^11^C-labelled erlotinib for visualization of EGFRs. Labelling of gefitinib with ^11^C (Wang *et al*, 2006; Kawamura *et al*, 2009; Zhang *et al*, 2010) and ^18^F (Su *et al*, 2008) has been attempted but ^18^F-gefitinib showed a high non-specific cellular uptake both *in vitro* and in mice xenografted with human tumours. Furthermore, in this *in-vivo* model the ^18^F-gefitinib signal did not relate to EGFR expression (Su *et al*, 2008). In contrast, ^11^C-gefitinib showed enhanced accumulation *in vitro* in the cancer cells that had the highest EGFR expression (Zhang *et al*, 2010). In a recent micro-PET study, we reported the development of a new radiotracer, ^11^C-erlotinib, and its use in mice models of human lung cancer (Memon *et al*, 2009). Our results showed that ^11^C-erlotinib accumulated in xenografts that were sensitive to erlotinib treatment and expressed high levels of EGFR.

In the present study in patients with NSCLC, we examined the feasibility of using ^11^C-erlotinib PET combined with X-ray computed tomography (CT) for visualization of tumour tissue, metastases, and malignant lymph nodes.

## Subjects and Methods

### Subjects and recruitment

Thirteen patients with NSCLC were included between December 2008 and October 2009 before start on second-line treatment with erlotinib. According to clinical practice, patients with metastatic lung cancer were treated with first-line chemotherapy as a palliative treatment (*n*=10) and patients with locally advanced lung tumours received a potentially curative treatment with concomitant chemotherapy and radiotherapy (*n*=3). If the patients progressed within 6 months as assessed by the ‘Response evaluation criteria in solid tumours’ (RECIST) (Eisenhauer *et al*, 2009) using contrast-enhanced CT, treatment with erlotinib was offered as second-line treatment.

Patients were eligible if they were over 18 years of age, had normal liver and kidney function as judged from blood tests, were non-diabetic, and could lie in the PET/CT scanner for 90 min (Table 1). Patients were excluded if they were allergic to X-ray contrast agent or had marked dyspnoea at rest. The study was approved by the Central Denmark Region Committees on Biomedical Research Ethics (M-20080050) and the Danish Medical Association (2512-96464) and conducted in accordance with the Helsinki II Declaration.

### Study design

The patients were examined by contrast-enhanced CT before inclusion into the study, as mentioned above. After inclusion, patients underwent pre-treatment ^11^C-erlotinib PET and ^18^F-FDG PET examinations combined with low-dose CT scans (scan 1). Erlotinib treatment was started immediately hereafter. Twelve weeks after start of the treatment, ^18^F-FDG PET combined with contrast-enhanced CT (scan 2) was performed. In patient no. 12, who discontinued the treatment after 7 weeks, scan 2 was performed at this time. The primary end point was higher accumulation of ^11^C-erlotinib in localized foci than in surrounding tissues and the secondary end point was clinical response, as assessed after 12 weeks of erlotinib treatment by the RECIST criteria and ^18^F-FDG PET/CT scans, or death of the patient.

The evaluation of the CT images was performed by an experienced radiologist and a nuclear medicine specialist and the evaluation of the PET images was performed by two nuclear medicine specialists; all assessors were blinded for other imaging data and the clinical status of the patient.

### Radiosynthesis of ^11^C-erlotinib

Erlotinib was labelled as described previously (Memon *et al*, 2009). Analytical HPLC showed the product to have >98% radiochemical purity with a specific activity of 5–200 GBq *μ*mol^−1^; it contained no compounds except for the precursor and the product (0.1–0.2 *μ*g ml^−1^) as determined by UV measurements. The product solution was clear and colourless with pH 5.5–6.5.

### Contrast-enhanced CT

Contrast-enhanced CT of the thorax was performed using a Philips, Brilliance 64 CT scanner (Eindhoven, The Netherlands) with 2 ml kg^−1^ body weight of intravenous contrast agent (Visipaque) containing 270 mg of iodine per millilitre.

### ^11^C-erlotinib PET/CT and ^18^F-FDG PET/CT

The patients were asked to fast overnight but were free to drink water. The patient was placed on the back in a 40-slice Siemens Biograph TruePoint PET/CT camera with a 21-cm transaxial field-of-view (Siemens AG, Erlangen, Germany). A low-dose CT scan (50 effective mAs with CAREDose4D, 120 kV, pitch 0.8, slice thickness 5 mm) was performed for definition of anatomical structures and attenuation correction of the PET recordings.

For the ^11^C-erlotinib PET/CT study, 500 MBq±10% ^11^C-erlotinib was administered intravenously and immediately followed by a dynamic PET recording of the thorax for 90 min using list-mode data acquisition. Two hours after the ^11^C-erlotinib administration (six times the radioactive half-life for ^11^C of 20 min), 400 MBq±10% ^18^F-FDG was administered intravenously and after 1 h, a static PET recording was performed from head to groin using list-mode acquisition. Images were reconstructed by an iterative algorithm (6 iterations, 14 subsets) resulting in 3-dimensional images consisting of 168 × 168 × 73 voxels of 4.0 × 4.0 × 3.0 mm^3^ followed by a post-reconstruction smoothing Gaussian filter (5-mm full-width at half-maximum).

Data from the dynamic ^11^C-erlotinib PET recordings following the initial 5 min vascular phase were summed to give images of the average radioactivity concentrations. Foci with radioactivity concentrations higher than in the surrounding tissue were defined as *hotspots* of lung tumours, lymph-node metastases, or distant metastases. Volumes-of-interest were drawn in the *hotspots* and in the normal lung and muscle tissue for extraction of time courses of radioactivity concentrations.

For ^18^F-FDG PET/CT, *hotspots* were defined as foci with increased activity concentration compared with the surrounding tissues as previously described (Fischer *et al*, 2009).

### Assessment of treatment response

Treatment response was assessed by comparison of pre-treatment (13–34 days before start of treatment, median 20 days) and post-treatment (12 weeks after start of treatment) contrast-enhanced CT images, using the CT-based tumour-node metastasis (TNM) staging system for lung cancer (Sobin and Fleming, 1997), the RECIST criteria, and ^18^F-FDG PET/CT. Patients having stable disease at the evaluation were classified as responders (*n*=5), whereas the non-responders consisted of the patients with progressive disease and the patients who died before the final assessment (*n*=8).

## Results

Of the 13 patients included, 5 patients had stable disease at the post-treatment assessment, 4 patients had progressive disease, and 4 patients had died (Table 2). Among the five patients with stable disease, three had ^11^C-erlotinib *hotspots* (Table 2). One patient (patient no. 7) with erlotinib *hotspot* died 5 days after the start of treatment due to liver failure. Patient no. 12 discontinued treatment after 7 weeks due to severe side effects (fatigue and diarrhoea, grade 3 based on Common Terminology Criteria for Adverse Events v3.0 (Cancer Therapy Evaluation Program, Common Terminology Criteria for Adverse Events, Version 3.0, DCTD, NCI, NIH, DHHS (31 March 2003) (http://ctep.cancer.gov), 9 August 2006)). Among the nine patients with no ^11^C-erlotinib *hotspots*, three died before evaluation, four showed progressive disease, while two had stable disease at the post-treatment assessment (Table 2).

**Figure 1:**
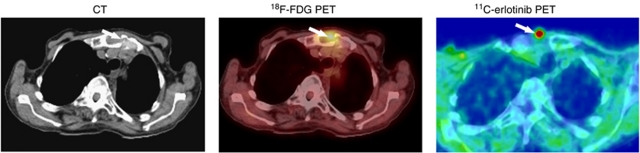
Accumulation of ^11^C-erlotinib in a bone metastasis from a NSCLC. Left: transaxial slices of contrast-enhanced CT; middle: ^18^F-FDG PET/low-dose CT; right: ^11^C-erlotinib PET/low-dose CT. A 79-year-old patient (no. 12) had NSCLC with metastasis to the sternum as shown on CT (arrow left figure). Both ^18^F-FDG and ^11^C-erlotinib accumulated in the metastatic lesion (arrows middle and right figures).

Figure 1 exemplifies CT, ^18^F-FDG PET/CT, and ^11^C-erlotinib PET/CT of a metastasis located in the sternum in a patient (no. 12) who responded to the treatment.

**Figure 2:**
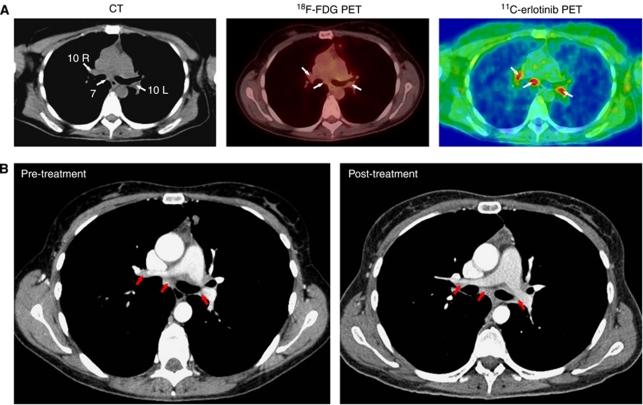
^11^C-erlotinib accumulation in lymph nodes that were negative on ^18^F-FDG PET/CT in a 42-year-old patient (no. 6). (**A**) Left: transaxial slices of contrast-enhanced CT; middle: ^18^F-FDG PET/low-dose CT; right: ^11^C-erlotinib PET/low-dose CT. CT (left figure) showed an enlarged lymph node (>10 mm) at position 7 (arrow) and non-enlarged lymph nodes (<10 mm) at positions 10R and 10L (arrows). None of these lymph nodes were visualized by ^18^F-FDG PET/CT (arrows, middle figure), whereas both enlarged and non-enlarged lymph nodes were visualized by ^11^C-erlotinib PET/CT (arrows, right figure). The ratio between ^11^C-erlotinib average radioactivity concentrations in the lymph nodes and that in surrounding lung tissue was 2. (**B**) Comparison of pre-treatment and 1-year post-treatment CT scans showed no significant change in the size of any of these lymph nodes.

Patient no. 6, who also responded to the treatment, CT showed non-enlarged (<10 mm) hilar lymph nodes (positions 10R and 10L) and an enlarged (>10 mm) subcarinal lymph node (position 7) (Figure 2A). There was no accumulation of ^18^F-FDG in any of these lymph nodes, whereas ^11^C-erlotinib PET/CT showed accumulation in both the enlarged and the non-enlarged lymph nodes (Figure 2A). The patient continued treatment after end of the study and at follow-up 1 year later, ^18^F-FDG PET/CT was negative and contrast-enhanced CT showed no changes in the size of these three lymph nodes (Figure 2B).

**Figure 3:**
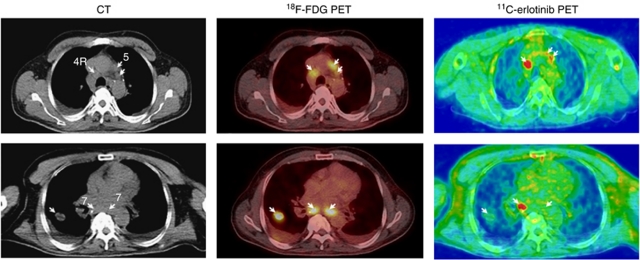
^11^C-erlotinib PET/CT demonstrates the heterogeneous nature of advanced lung cancer. Two transaxial slices of (left) contrast-enhanced CT; (middle) ^18^F-FDG PET/low-dose CT, and (right) ^11^C-erlotinib PET/low-dose CT. A 48-year-old patient (no. 7) with NSCLC in the right lung and enlarged mediastinal lymph nodes (upper and lower panel left figure, arrows). Both ^18^F-FDG and ^11^C-erlotinib accumulated in lymph nodes at positions 4R and 5 (upper panel, arrows). The tumour in the right lung and one of the lymph nodes at position 7 showed only a weak accumulation of ^11^C-erlotinib (arrows). The ratio between the ^11^C-erlotinib average radioactivity concentrations in the lymph node metastasis and that in surrounding lung tissue was 2 (see Figure 4).

**Figure 4:**
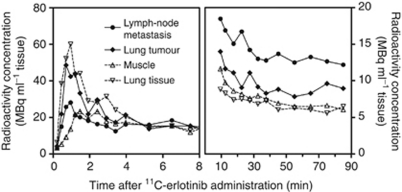
Time courses of tissue radioactivity of ^11^C-erlotinib (patient no. 7, see Figure 3) for the right-sided lung tumour, metastatic lymph node 4R, lung tissue, and muscle tissue. Left: initial time courses and right: final time courses. The final accumulation of ^11^C-erlotinib was higher in the tumour and the metastatic lymph node than in lung and muscle tissue.

Patient no. 7 showed different tumour foci that differed with regard to accumulation of ^11^C-erlotinib as illustrated in Figure 3. Enlarged lymph nodes in the mediastinum at positions 4R and 5 (CT, arrows) showed *hotspots* on ^18^F-FDG PET/CT and on ^11^C-erlotinib PET/CT (upper panel, arrows) whereas the lung tumour and one of the enlarged lymph nodes at position 7 did not accumulate ^11^C-erlotinib (lower panel, arrows). Time courses of the radioactivity concentrations of ^11^C-erlotinib in this patient (Figure 4) show higher initial distribution in lung tissue and in the lung tumour than in the subcarinal lymph node and muscle tissue (Figure 4, left), which can be ascribed to differences in the blood perfusion. Ten minutes after the ^11^C-erlotinib administration and throughout the remaining 80-min measurement period, the radioactivity concentration was higher in the lymph node and the lung tumour than in the non-malignant lung and muscle tissue (Figure 4, right). The ratio between radioactivity concentrations lung tumour/lung tissue was around 1.3 and that of the lymph node/lung tissue around 2.0.

## Discussion

In total, four of the thirteen patients examined showed ^11^C-erlotinib accumulation in one or more tumour foci and ^11^C-erlotinib accumulated in non-enlarged ^18^F-FDG PET/CT-negative lymph nodes. Our results also showed variation in ^11^C-erlotinib accumulation between different malignant lesions in the same patient.

Erlotinib is a targeted drug that inhibits signalling through the EGFR and thereby prolongs survival of a subgroup of lung cancer patients treated with this drug. Clinical parameters and mutational status of EGFR are considered helpful but insufficient to predict treatment response; and therefore, additional methods are required to improve the selection of patients for erlotinib treatment. We previously showed that ^11^C-erlotinib could be used to identify tumours overexpressing the EGFR in animal models (Memon *et al*, 2009), and we have presented a case report showing accumulation of ^11^C-erlotinib in brain metastasis of a patient with NSCLC (Weber *et al*, 2011). Here, we investigated the use of ^11^C-erlotinib PET/CT as a non-invasive method to identify tumours accumulating erlotinib and have established a method that may prove useful for the selection of patients suitable for erlotinib treatment. Our results showed that normal lung tissue accumulated no or minimal ^11^C-erlotinib, whereas some malignant lesions and metastases did accumulate the tracer.

Because tumour material was not available from all lesions, the EGFR status in ^11^C-erlotinib accumulating and non-accumulating tumour lesions is not known. However, in the present study, we observed different accumulation of ^11^C-erlotinib in the primary tumour and metastatic lesions in the same patient. This suggests that it may be of limited value to know the EGFR status in a biopsy from just one tumour lesion.

Variation in the accumulation of ^11^C-erlotinib between different tumours in the same patient is in agreement with the molecular evolution of the disease in these patients. The majority of lung cancer patients who initially respond to treatment eventually have a relapse (Sequist *et al*, 2007). Some of the known causes of these relapses are mutations in EGFR causing erlotinib resistance, amplification of the met proto-oncogene (MET) (Engelman *et al*, 2007), activation of other receptor tyrosine kinases (insulin-like growth factor 1) (Morgillo *et al*, 2007), and *KRAS* mutations (Eberhard *et al*, 2005). Our observations of variation in the uptake of ^11^C-erlotinib further suggest that not all tumours in the same patient are driven by EGFR signalling, and therefore may not respond to erlotinib treatment.

Staging of lung cancer is crucial when deciding treatment options and the prognosis also differs significantly according to stage. The TNM preoperative staging system employing CT is widely used; however, lymph-node staging (N staging) of hilar and mediastinal lymph nodes is still a challenge and CT scanning results in a significant amount of false positive and false negative results (Al-Sarraf *et al*, 2008). Another method for staging is ^18^F-FDG PET/CT (Fischer *et al*, 2009). The major obstacle with this method is the difficulty in distinguishing between benign and metastatic lymph nodes (Love *et al*, 2005). According to standard CT criteria, lymph nodes with a diameter >10 mm (enlarged) are classified as malignant and lymph nodes with a diameter <10 mm (non-enlarged) are classified as non-malignant. However, recent studies suggest that non-enlarged lymph nodes can also be metastatic even in the absence of a positive ^18^F-FDG PET/CT and small lymph nodes can therefore not be identified as non-malignant until other characteristics are considered (Liu *et al*, 2009). Interestingly, ^11^C-erlotinib PET/CT identified both enlarged and non-enlarged lymph nodes, which were negative on ^18^F-FDG PET/CT. These results indicate that it is possible that non-enlarged lymph nodes in cancer patients may harbour tumour cells expressing EGFR. We monitored these lymph nodes during erlotinib treatment in patient no. 6, and both enlarged and non-enlarged lymph nodes remained stable according to the RECIST criteria for more than a year and were also negative on ^18^F-FDG PET/CT. If these findings are confirmed in more patients, then this observation could have clinical significance as it may change the criteria for staging of lung cancer and thereby the treatment strategy for the patients.

## Conclusions

This study showed a potential for ^11^C-erlotinib in PET/CT for visualizing NSCLC lung tumours, including lymph nodes not identified by ^18^F-FDG PET/CT. Moreover, ^11^C-erlotinib in PET/CT could be a useful tool to identify molecular heterogeneity between tumours in the same patient. Large clinical studies are now needed to explore to which extent pre-treatment ^11^C-erlotinib PET/CT can predict response to erlotinib treatment.

## Figures and Tables

**Table 1:** Clinical characteristics of patients with non-small lung cancer (NSCLC) at inclusion for ^11^C-erlotinib PET/CT

*Patients (n)*	13
Male	4
Female	9
Age (years) (median; range)	63 (42–79)
	
*Histology of NSCLC (n)*	
Adenocarcinoma	8
Squamous cell carcinoma	3
Large-cell carcinoma	1
Unspecified	1
	
*TNM classification (n)*	
Stage	
Tx	2
T1	2
T2	4
T3–4	5
Node	
Nx	1
N1	0
N2	7
N3	5
Metastasis	
M0	2
M1A	0
M1B	11

Abbreviations: CT=computed tomography; PET=positron emission tomography; *n*=number of patients; TNM=tumour-node metastasis.

**Table 2:** Clinical parameters related to erlotinib treatment, status of ^11^C-erlotinib PET/CT, and clinical response after 12 weeks erlotinib treatment

**Patient no.^a^**	**Gender**	**Smoking status**	**Histology**	**^11^C-erlotinib *hotspot***	**Treatment response**
6	F	Never smoked	Adeno	Yes	SD
8	F	Never smoked	Adeno	Yes	SD
12	F	Never smoked	Adeno	Yes	SD^b^
7	M	Never smoked	Adeno	Yes	—c,^d^
5	F	Former smoker	SCC	No	SD
11	F	Former smoker	Adeno	No	SD
2	F	Former smoker	Adeno	No	PD
3	F	Former smoker	Not specified	No	PD
9	M	Former smoker	Adeno	No	PD
10	M	Active smoker	SCC	No	PD
1	M	Never smoked	Adeno	No	—d
4	F	Former smoker	Large-cell carcinoma	No	—d
13	F	Former smoker	SCC	No	—d

Abbreviations: Adeno=adenocarcinoma; CT=computed tomography; PD=progressive disease; PET=positron emission tomography; SCC=squamous-cell carcinoma; SD=stable disease.

a No. indicates the ID of the patients and was assigned as the patient was included in the study.

b Patient discontinued treatment after 7 weeks because of severe side effects.

c Patient died 5 days after start of erlotinib treatment.

d Patient died within study period.

## References

[bib1] Al-Sarraf N, Aziz R, Gately K, Lucey J, Wilson L, McGovern E, Young V (2008) Pattern and predictors of occult mediastinal lymph node involvement in non-small cell lung cancer patients with negative mediastinal uptake on positron emission tomography. Eur J Cardiothorac Surg 33: 104–109 17977738 10.1016/j.ejcts.2007.09.026

[bib2] Ciardiello F, Tortora G (2008) EGFR antagonists in cancer treatment. N Engl J Med 358: 1160–1174 18337605 10.1056/NEJMra0707704

[bib3] Cohen MH, Johnson JR, Chen YF, Sridhara R, Pazdur R (2005) FDA drug approval summary: erlotinib (Tarceva) tablets. Oncologist 10: 461–466 16079312 10.1634/theoncologist.10-7-461

[bib4] Comis RL (2005) The current situation: erlotinib (Tarceva) and gefitinib (Iressa) in non-small cell lung cancer. Oncologist 10: 467–470 16079313 10.1634/theoncologist.10-7-467

[bib5] Eberhard DA, Johnson BE, Amler LC, Goddard AD, Heldens SL, Herbst RS, Ince WL, Janne PA, Januario T, Johnson DH, Klein P, Miller VA, Ostland MA, Ramies DA, Sebisanovic D, Stinson JA, Zhang YR, Seshagiri S, Hillan KJ (2005) Mutations in the epidermal growth factor receptor and in KRAS are predictive and prognostic indicators in patients with non-small-cell lung cancer treated with chemotherapy alone and in combination with erlotinib. J Clin Oncol 23: 5900–5909 16043828 10.1200/JCO.2005.02.857

[bib6] Eisenhauer EA, Therasse P, Bogaerts J, Schwartz LH, Sargent D, Ford R, Dancey J, Arbuck S, Gwyther S, Mooney M, Rubinstein L, Shankar L, Dodd L, Kaplan R, Lacombe D, Verweij J (2009) New response evaluation criteria in solid tumours: revised RECIST guideline (version 1.1). Eur J Cancer 45(2): 228–247 19097774 10.1016/j.ejca.2008.10.026

[bib7] Engelman JA, Zejnullahu K, Mitsudomi T, Song Y, Hyland C, Park JO, Lindeman N, Gale CM, Zhao X, Christensen J, Kosaka T, Holmes AJ, Rogers AM, Cappuzzo F, Mok T, Lee C, Johnson BE, Cantley LC, Janne PA (2007) MET amplification leads to gefitinib resistance in lung cancer by activating ERBB3 signaling. Science 316: 1039–1043 17463250 10.1126/science.1141478

[bib8] Fischer B, Lassen U, Mortensen J, Larsen S, Loft A, Bertelsen A, Ravn J, Clementsen P, Hogholm A, Larsen K, Rasmussen T, Keiding S, Dirksen A, Gerke O, Skov B, Steffensen I, Hansen H, Vilmann P, Jacobsen G, Backer V, Maltbaek N, Pedersen J, Madsen H, Nielsen H, Hojgaard L (2009) Preoperative staging of lung cancer with combined PET-CT. N Engl J Med 361: 32–39 19571281 10.1056/NEJMoa0900043

[bib9] Fontanini G, De Laurentiis M, Vignati S, Chine S, Lucchi M, Silvestri V, Mussi A, De Placido S, Tortora G, Bianco AR, Gullick W, Angeletti CA, Bevilacqua G, Ciardiello F (1998) Evaluation of epidermal growth factor-related growth factors and receptors and of neoangiogenesis in completely resected stage I-IIIA non-small-cell lung cancer: amphiregulin and microvessel count are independent prognostic indicators of survival. Clin Cancer Res 4: 241–249 9516978

[bib10] Fukuoka M, Yano S, Giaccone G, Tamura T, Nakagawa K, Douillard JY, Nishiwaki Y, Vansteenkiste J, Kudoh S, Rischin D, Eek R, Horai T, Noda K, Takata I, Smit E, Averbuch S, Macleod A, Feyereislova A, Dong RP, Baselga J (2003) Multi-institutional randomized phase II trial of gefitinib for previously treated patients with advanced non-small-cell lung cancer. J Clin Oncol 21: 2237–2246 12748244 10.1200/JCO.2003.10.038

[bib11] Gambhir SS (2002) Molecular imaging of cancer with positron emission tomography. Nat Rev Cancer 2: 683–693 12209157 10.1038/nrc882

[bib12] Jerusalem G, Hustinx R, Beguin Y, Fillet G (2003) PET scan imaging in oncology. Eur J Cancer 39: 1525–1534 12855258 10.1016/s0959-8049(03)00374-5

[bib13] Kawamura K, Yamasaki T, Yui J, Hatori A, Konno F, Kumata K, Irie T, Fukumura T, Suzuki K, Kanno I, Zhang MR (2009) *In vivo* evaluation of P-glycoprotein and breast cancer resistance protein modulation in the brain using [^11^C]gefitinib. Nucl Med Biol 36: 239–246 19324269 10.1016/j.nucmedbio.2008.12.006

[bib14] Liu BJ, Dong JC, Xu CQ, Zuo CT, Le JJ, Guan YH, Zhao J, Wu JF, Duan XH, Cao YX (2009) Accuracy of ^18^F-FDG PET/CT for lymph node staging in non-small-cell lung cancers. Chin Med J (Engl) 122: 1749–1754 19781319

[bib15] Love C, Tomas MB, Tronco GG, Palestro CJ (2005) FDG PET of infection and inflammation. Radiographics 25: 1357–1368 16160116 10.1148/rg.255045122

[bib16] Lynch TJ, Bell DW, Sordella R, Gurubhagavatula S, Okimoto RA, Brannigan BW, Harris PL, Haserlat SM, Supko JG, Haluska FG, Louis DN, Christiani DC, Settleman J, Haber DA (2004) Activating mutations in the epidermal growth factor receptor underlying responsiveness of non-small-cell lung cancer to gefitinib. N Engl J Med 350: 2129–2139 15118073 10.1056/NEJMoa040938

[bib17] Memon AA, Jakobsen S, Dagnaes-Hansen F, Sorensen BS, Keiding S, Nexo E (2009) Positron emission tomography (PET) imaging with [^11^C]-labeled erlotinib: a micro-PET study on mice with lung tumor xenografts. Cancer Res 69: 873–878 19155297 10.1158/0008-5472.CAN-08-3118

[bib18] Morgillo F, Kim WY, Kim ES, Ciardiello F, Hong WK, Lee HY (2007) Implication of the insulin-like growth factor-IR pathway in the resistance of non-small cell lung cancer cells to treatment with gefitinib. Clin Cancer Res 13: 2795–2803 17473213 10.1158/1078-0432.CCR-06-2077

[bib19] Paez JG, Janne PA, Lee JC, Tracy S, Greulich H, Gabriel S, Herman P, Kaye FJ, Lindeman N, Boggon TJ, Naoki K, Sasaki H, Fujii Y, Eck MJ, Sellers WR, Johnson BE, Meyerson M (2004) EGFR mutations in lung cancer: correlation with clinical response to gefitinib therapy. Science 304: 1497–1500 15118125 10.1126/science.1099314

[bib20] Parkin DM, Bray F, Ferlay J, Pisani P (2005) Global cancer statistics, 2002. CA Cancer J Clin 55: 74–108 15761078 10.3322/canjclin.55.2.74

[bib21] Perez-Soler R, Chachoua A, Hammond LA, Rowinsky EK, Huberman M, Karp D, Rigas J, Clark GM, Santabarbara P, Bonomi P (2004) Determinants of tumor response and survival with erlotinib in patients with non-small-cell lung cancer. J Clin Oncol 22: 3238–3247 15310767 10.1200/JCO.2004.11.057

[bib22] Rohren EM, Turkington TG, Coleman RE (2004) Clinical applications of PET in oncology. Radiology 231: 305–332 15044750 10.1148/radiol.2312021185

[bib23] Rusch V, Baselga J, Cordon-Cardo C, Orazem J, Zaman M, Hoda S, McIntosh J, Kurie J, Dmitrovsky E (1993) Differential expression of the epidermal growth factor receptor and its ligands in primary non-small cell lung cancers and adjacent benign lung. Cancer Res 53: 2379–2385 7683573

[bib24] Sequist LV, Bell DW, Lynch TJ, Haber DA (2007) Molecular predictors of response to epidermal growth factor receptor antagonists in non-small-cell lung cancer. J Clin Oncol 25: 587–595 17290067 10.1200/JCO.2006.07.3585

[bib25] Shepherd FA, Pereira J, Ciuleanu TE, Tan EH, Hirsh V, Thongprasert S, Bezjak A, Tu D, Santabarbara P, Seymour L (2004) A randomized placebo-controlled trial of erlotinib in patients with advanced non-small cell lung cancer (NSCLC) following failure of 1(st) line or 2(nd) line chemotherapy. A National Cancer Institute of Canada Clinical Trials Group (NCIC CTG) trial. J Clin Oncol 22: 622S

[bib26] Shepherd FA, Pereira JR, Ciuleanu T, Tan EH, Hirsh V, Thongprasert S, Campos D, Maoleekoonpiroj S, Smylie M, Martins R, van Kooten M, Dediu M, Findlay B, Tu DS, Johnston D, Bezjak A, Clark G, Santabarbara P, Seymour L (2005) Erlotinib in previously treated non-small-cell lung cancer. N Engl J Med 353: 123–132 16014882 10.1056/NEJMoa050753

[bib27] Sobin LH, Fleming ID (1997) TNM classification of malignant tumors, fifth edition (1997). Union Internationale Contre le Cancer and the American Joint Committee on Cancer. Cancer 80: 1803–1804 9351551 10.1002/(sici)1097-0142(19971101)80:9<1803::aid-cncr16>3.0.co;2-9

[bib28] Su H, Seimbille Y, Ferl GZ, Bodenstein C, Fueger B, Kim KJ, Hsu YT, Dubinett SM, Phelps ME, Czernin J, Weber WA (2008) Evaluation of [^18^F]gefitinib as a molecular imaging probe for the assessment of the epidermal growth factor receptor status in malignant tumors. Eur J Nucl Med Mol Imaging 35: 1089–1099 18239919 10.1007/s00259-007-0636-6

[bib29] Wang JQ, Gao M, Miller KD, Sledge GW, Zheng QH (2006) Synthesis of [11C]Iressa as a new potential PET cancer imaging agent for epidermal growth factor receptor tyrosine kinase. Bioorg Med Chem Lett 16: 4102–4106 16697188 10.1016/j.bmcl.2006.04.080

[bib30] Weber B, Winterdahl M, Memon A, Sorensen BS, Keiding S, Sorensen L, Nexo E, Meldgaard P (2011) Erlotinib accumulation in brain metastases from non-small cell lung cancer: visualization by positron emission tomography in a patient harboring a mutation in the epidermal growth factor receptor. J Thorac Oncol 6: 1287–1289 21847041 10.1097/JTO.0b013e318219ab87

[bib31] Zhang MR, Kumata K, Hatori A, Takai N, Toyohara J, Yamasaki T, Yanamoto K, Yui J, Kawamura K, Koike S, Ando K, Suzuki K (2010) [^11^C]Gefitinib ([^11^C]Iressa): radiosynthesis, *in vitro* uptake, and *in vivo* imaging of intact murine fibrosarcoma. Mol Imaging Biol 12: 181–191 19784702 10.1007/s11307-009-0265-5

